# Allosteric Regulation of Serine Protease HtrA2 through Novel Non-Canonical Substrate Binding Pocket

**DOI:** 10.1371/journal.pone.0055416

**Published:** 2013-02-14

**Authors:** Pruthvi Raj Bejugam, Raja R. Kuppili, Nitu Singh, Nikhil Gadewal, Lalith K. Chaganti, G. Madhavi Sastry, Kakoli Bose

**Affiliations:** 1 Advanced Centre for Treatment, Research and Education in Cancer (ACTREC), Tata Memorial Centre, Kharghar, Navi Mumbai, India; 2 Schrödinger, Sanali Infopark, Banjara Hills, Hyderabad, India; IISER-TVM, India

## Abstract

HtrA2, a trimeric proapoptotic serine protease is involved in several diseases including cancer and neurodegenerative disorders. Its unique ability to mediate apoptosis via multiple pathways makes it an important therapeutic target. In HtrA2, C-terminal PDZ domain upon substrate binding regulates its functions through coordinated conformational changes the mechanism of which is yet to be elucidated. Although allostery has been found in some of its homologs, it has not been characterized in HtrA2 so far. Here, with an *in silico* and biochemical approach we have shown that allostery does regulate HtrA2 activity. Our studies identified a novel non-canonical selective binding pocket in HtrA2 which initiates signal propagation to the distal active site through a complex allosteric mechanism. This non-classical binding pocket is unique among HtrA family proteins and thus unfolds a novel mechanism of regulation of HtrA2 activity and hence apoptosis.

## Introduction

Multidomain proteins due to their structural complexity require different levels of regulatory mechanisms for executing cellular functions efficiently within a specified time period. Allosteric modulation of conformations is one such mechanism which often helps a protein to regulate a functional behaviour such as for an enzyme to attain an active functional state upon ligand or substrate binding. In allostery, sometimes there are large conformational changes that require significant rotations and translations of individual domains at the timescales of microsecond to millisecond. While in some other cases, minimal structural perturbation helps in propagation of the signal in an energy efficient way to the functional domain where movement is mainly restricted to the side chains, loops and linker regions and which occur within picosecond to nanosecond timescales [1]. PDZ (post-synaptic density-95/discs large/zonula occludens-1) domains that are involved in myriads of protein-protein interactions [2], [3] exhibit minimal structural changes during allosteric propagation. These domains have multiple ligand docking sites and are known to possess unique dynamics that regulate conformation of the functional site from a distal region.

HtrA2 (High temperature requirement protease A2), a PDZ bearing protein, is a mitochondrial trimeric pyramidal proapoptotic serine protease with complex domain architecture whose activity is likely regulated by interdomain crosstalk and structural plasticity [4]. Mature HtrA2 comprises 325 amino acids with residues S173, D95 and H65 forming the catalytic triad which is buried 25 Å above the base of the pyramid suggesting requirement of conformational changes for its activation. Apart from PDZ, this multidomain protein has a short N-terminal region, a serine protease domain and a non-conserved flexible linker at the PDZ- protease interface [4]. HtrA2 is involved in both caspase dependent as well as caspase independent apoptotic pathways [5], [6], [7]. Literature suggests it might have chaperoning functions as well and recently has been found to be associated with several neurodegenerative disorders [8], [9], [10]. Based on information from literature [4], [11], this multitasking ability of HtrA2 can be attributed to its serine protease activity which is intricately coordinated by its unique substrate binding process, complex trimeric structure, interdomain networking and conformational plasticity. However, the unbound inactive form of the crystal structure with partially missing active site loops and flexible PDZ-protease linker has been unable to unambiguously determine the role of dynamics and allostery if any in HtrA2 activation and specificity. Therefore, to understand the molecular details of its mechanism of action, dynamics study at the substrate binding site and active site pocket becomes imperative.

HtrA2 belongs to a serine protease family that is conserved from prokaryotes to humans [12] where allostery is a common mechanism for protease activation in some of its homologs. DegS, a bacterial counterpart of HtrA2, allosterically stabilizes the active site pocket upon substrate binding at the distal PDZ domain [13]. DegP, the most extensively studied protein of the family, has a cage-like hexameric structure whose activation is regulated by allostery and oligomerization. Peptide binding to distal PDZ1 domain leads to rearrangement of the catalytic pocket into enzymatically competent form that readily oligomerizes and renders stability to the active conformation [14].

With an aim at understanding the conformational changes and structural plasticity that govern HtrA2 activity and specificity, we took an *in silico* approach to study the movements of flexible regions of the protein upon ligand binding. The PDZ domain of HtrA2 has a known hydrophobic substrate binding YIGV pocket (similar to GLGF motif) which is deeply embedded within the trimeric protein structure with P225 and V226 from the serine protease domain occupying the groove [4], [15]. This structural arrangement makes it impossible for substrate protein to bind without significant conformational changes. Thus, to examine whether allosteric modulation through an alternative site is involved in substrate binding and catalysis of HtrA2, molecular dynamics simulation (MDS) approach with a bound peptide activator was used to look into the structural rearrangements that occur in nanosecond time scale. Although the information usually obtained from MDS is restricted primarily to movements in the accessible and flexible regions of a protein, it nonetheless contributes significantly towards understanding of the overall structural rearrangement and dynamics during its allosteric activation. In our study, we modelled the entire mature protease by filling in the missing regions using Prime 3.0 [16], followed by energy minimization with GRoningen MAchine for Chemical Simulation or GROMACS [17]. Identification of the putative binding site(s) on HtrA2 was done using SiteMap 2.5 [18] and the selective binding pocket (SBP) for the ligand was chosen based on optimum energy parameters. Peptides at SBP were docked from our peptide library that was generated based on available literature reports [19], [20], [21] and structural complementarities. MDS of the docked structures was done using Desmond 2010 [22] which provided critical information on loop and linker movements in HtrA2. These results combined with mutational and enzymology studies show that upon activator binding at the novel allosteric pocket, SBP, the linker at the PDZ-protease interface and loops L1, LA and LD around the catalytic groove undergo rearrangements in a coordinated manner so as to form an efficient active site pocket. Moreover, the PDZ domains mediate intersubunit interactions which stabilize the oxyanion hole. These observations highlight the importance of allostery which might be an important prerequisite for an active conformation of the trimeric protease.

## Results

### Identification of Selective Binding Pocket (SBP)

**Figure 1:**
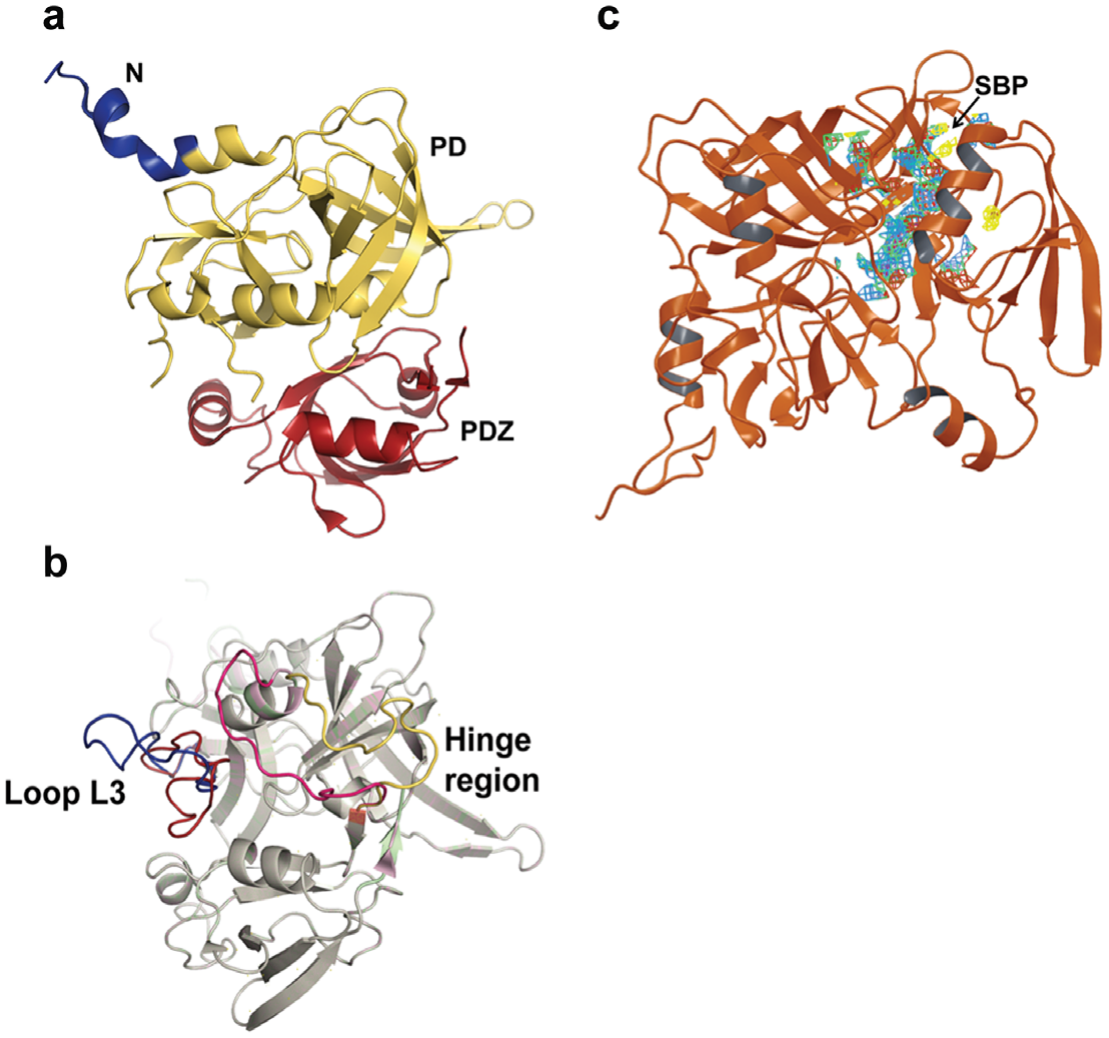
Ribbon model of HtrA2 structures (PDB ID: 1LCY). a. Domain organization of HtrA2 protease which comprises N-terminal region (blue), protease domain denoted as PD (yellow) and PDZ domain (red) at C-terminal end. b. Structural alignment of loop refined (light magenta) and unrefined (light green) structures of HtrA2 protein with modelled N-terminal AVPS, loop L3 (residues 142–162) and hinge region (residues 211–225) built with Prime (Schrödinger 2011). On refinement, loop L3 and hinge region are reorganized so as to define new regions at the protease and PDZ domain interface. c. Selective binding pocket (SBP) on HtrA2. The energy minimised structure of HtrA2 after modelling flexible regions in the protein is represented as a ribbon model. The binding site designated as SBP, selected on the basis of the Sitemap score and residue analyses, is located at the interface of PDZ and protease domain and shown as a multi-coloured mesh.

The high resolution crystal structure of HtrA2 [4] (Figure 1a) that lacked flexible loops, linkers and some N-terminal residues was the target protein for our studies. These regions were modelled and energy minimised as described under Methods section. Comparison of refined model with unrefined structure showed significant movements of the loops defining new binding sites on the protein surface. The linker at SPD-PDZ interface moved towards α7 of PDZ domain whereas the linker in the protease domain moved closer to the SPD-PDZ linker so as to form a groove (Figure 1b).

Among the five possible putative binding sites that were identified, Site2 or SBP (Figure 1c) that encompasses the groove generated by SPD-PDZ linker, protease and PDZ domains attained the best score (Table 1). The site score takes into account parameters such as volume, density, solvent exposure, hydrophilic and hydrophobic nature of residues and donor to acceptor ratio and hence is a comprehensive representation of the possibility of it being a binding site.

**Table 1:** Putative binding sites in HtrA2 identified by SiteMap tool.

Site Number from SiteMap	Residues present in the site	Site score
Site 2	K214, K215, N216,S217,S219, R226, R227, Y228, I229, G230,V231,M232,M233, L234, T235, L236,S237, S239, I240, E243, H256, K262, I264,Q289, N290, A291,E292, Y295,E 296, R299, S302	1.092716
Site 1	H65, D69, R71, A89, V90, P92, D95,T324	0.957142
Site 3	N48, H65, D169, S173,K191, M232, H261,L265	0.936056
Site 4	V192, F251	0.807891
Site 5	I33,L34,D35,R36,V73,R74	0.673032

SBP has optimum volume and contacts available including maximum hydrogen donor and acceptor groups that are crucial for interacting with peptides. The size of the site is very important since the binding peptides have 6–7 residues and the site needs to be large enough to accommodate them. It also has highest hydrophobicity which makes it the best interaction site and hence used in our studies. Although sites 1 and 3 have scores closer to that of SBP, taking into account all the above-mentioned parameters, SBP was chosen for further docking and MDS studies.

### Peptide Docking Show Similar Interacting Residues

Here, we have used a holistic approach in designing activator peptides where different techniques were applied in parallel so as to conduct a comprehensive search for a signature pattern that would dock at SBP. In one method, replicas for functional groups were chosen based on sequence and structural complementarities with hydrophobic SBP which were used for generating small molecular fragments. Scores obtained from docking these small molecules (Table S1) provided the framework for designing different combinations of tetrapeptides as shown in Table S2. With leads from literature and *in silico* structure-guided design, Gly and Val residues were added at N- and C-termini respectively of some peptides which subsequently increased the docking scores from −6 to −10 kcal/mol.

Similarly, two peptides previously reported in the literature as well peptides designed from the putative binding sites in pea-15 and Hax-1 also interacted well with SBP. Analysis of docking results with all these different peptides show interaction with similar residues of SBP as observed in ligplot (Figure S1). However, the control peptide KNNPNNAHQN, which has quite a few asparagine residues, is an ideal sequence to act as negative peptide for the pocket due to its stereochemical properties [19], did not bind to SBP demonstrating the specificity of designed peptides.

**Figure 2:**
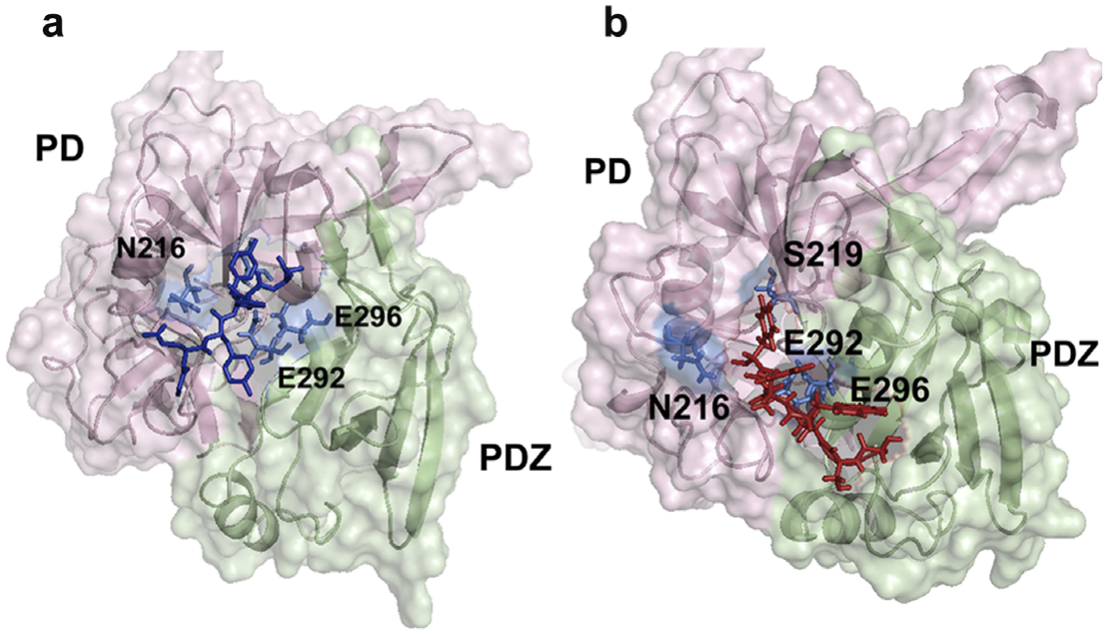
Representative surface structures of peptide activator docked HtrA2. a. Peptide GSAWFSF -HtrA2 complex and b. Peptide GQYYFV-HtrA2 complex. The former peptide represents putative SBP binding peptide in Pea-15 and the latter is a peptide obtained from the literature. The common interacting residues from SBP for both the peptides are labelled and are shown as blue sticks. PD denotes serine protease domain in both the Figures.

From the above extensive docking analysis, N216, S217, S219, E292 and E296 in SBP were found to be common for most of the peptide interactions (Figures 2a–b). Of these residues, N216, S217, S219 belong to the linker region while E292 and E296 to the PDZ domain that were either involved in hydrogen bond formation or Van der Waals interaction with the peptides. This result suggests that SBP might be the possible binding site and therefore a prospective putative allosteric site.

The role of some of these important residues in allostery if any and its subsequent effect on catalytic activity and substrate turnover was further probed by enzymology studies as described later in the text.

### MDS Analyses of HtrA2 and HtrA2– Peptide Complexes

**Figure 3:**
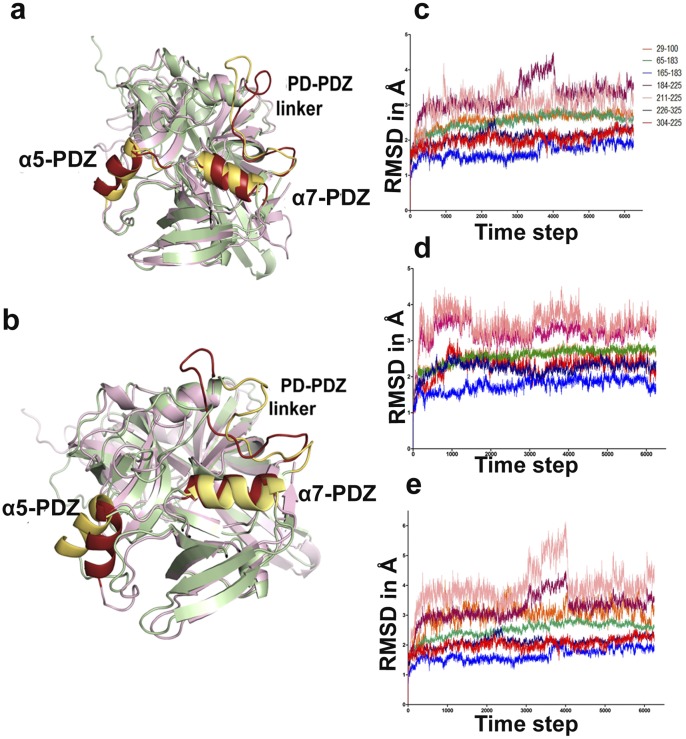
Domain wise conformational changes induced on peptide binding at SBP. a. The structural alignment of minimum energy structure of the peptide bound GQYYFV-HtrA2 complex (light pink) and unbound structure (green) displays orientation of the movement of the hinge region and the α-helices of PDZ. b. The structural alignment of GSAWFSF-HtrA2 complex (light pink) and unbound structure (green). Graphical representations of the RMSD for the 30 ns MDS trajectory of the following: c. HtrA2–GQYYFV complex. d. unbound HtrA2 (negative control). e. HtrA2–GSAWFSF complex. The stretch of residues selected for each set of RMSD calculations are shown on the right of panel c.

The peptides GSAWFSF was chosen for MDS studies as it gave the best XP and E-model scores (Table 2). GQYYFV has been reported to be a well known activator of HtrA2 [19] and hence used as another representative peptide for simulation studies. Moreover, the two peptides were chosen such that one is a designed peptide (GQYYFV) while the other is a part of a well-known HtrA2 binding protein Pea-15 (GSAWFSF). In addition to this, GQYYFV with docking score lesser than GSAWFSF was chosen for MDS analysis to understand whether different affinity for the substrate results in similar movements in the protease. MDS analyses of HtrA2-GQYYFV and HtrA2-GSAWFSF complexes demonstrated significant difference in conformation as well as dynamics when compared with unbound HtrA2. Visual inspection of the domain wise movements in peptide bound HtrA2 indicated large fluctuations in hinge/linker region (211–226) as shown in Figures 3a and b. Although these movements were larger for GSAWFSF than GQYYFV bound complex, the movement pattern remained similar in these two peptides. Enhanced dynamic movement in the former complex could be attributed to the peptide length (heptameric as compared to hexameric in the latter). Domain wise RMSD analysis of these trajectories provided quantitative output of deviations with respect to time. The trajectory graphs (Figures 3c–e) show that along the entire sequence, hinge region (211 −226) has RMSD of 2.5 Å for the peptide GSAWFSF and 1.5 Å for GQYYFV from the starting unbound form.

**Table 2:** Peptide docking of HtrA2 and identification of interacting residues.

Peptides Used in Our study	Interacting Residues		Glide score in Kcal.mole^−1^
	H bond Interactions	Vdw Interactions	
**PEA 15 (GSAWFSF)**	Glu 292, Glu 296, Asp 293, Ile 283, Met 287	Gln 286, Ala 297, Ser 222	−10.564
**Designed (VKSDSG)**	Asn 216, Leu 152,Glu 296, Glu 292	Ala 89, Ile 221, ser 218,	−10.394
**Designed (GRTDSV)**	Glu 296, Glu 292, Asn 216, Ser 217	Asp 293	−10.037
**Designed (GRDTSV)**	Ser 219, Glu 292	Ser 239,Gln 286	−9.57
**Designed (GRDTYV)**	Asp 293, Asn 216, Ser 217, Ser 219	Glu 296, Arg 299,	−9.54
**Phosphatase (PAEWTRY)**	Asp 117, Ala 149, Arg 150, Lys 215,Gln 146	Pro 148, Leu 152, Lys 214, Gln 156,Val 159, Ser 239	−9.481
**HAX-1 (TKPDIGV)**	Glu 292, Glu 296, Ser 219, Ile 221, Arg 299	Asn 216, Ser 222	−8.486
**Connexin (ARKSEWV)**	Asp 293/426, Asn 290/423, Gln 156/289	Glu 292, Pro 155, Gln 289, Met 287, His 256,Glu 255, pro 238	−8.165
**Presenilin (AFHQFYI)**	Leu 152, Asn 216, Ser 217, Glu 292,Glu 296	Pro 155, Arg 211, ser 218, Ser 219	−8.063
**IL-EBF (AGYTGFV)**	Asn 216, Ser 217, Glu 292, Arg 150/, Leu 152	Ser 219, Gly 153, pro 155	−7.903
**Yes Protein (ESFLTWL)**	Asn 216, Leu 152, Glu 296, Asp 293,Gln 289, Ser 237	Gln 156, Pro 238, Pro 155, Ser 218,Glu 292, Gln 286	−7.722
**Cathepsin SVSSIFV**	Glu 296, Asn 216, Ile 283/416,	Glu 292, Leu 152,Gly 153,Ala 297	−7.524
**Warts Protein Kinase (NRDLVYV)**	Lys 214, Lys215, Ala 149,Glu 207,Arg150, Gln 146	Leu 152, Gln 156, Val 159	−7.321
**GQYYFV** ^6^	Glu 292, Glu 296, Asn 216, Ile 221,Leu 152	Ser 219,Gly 153, Arg 299	−7.163
**GGIRRV** ^6^	Glu 292, Glu 296, Asn 216,Ser 217, Ser 219	Arg 211, Gly 153	−6.785
**Tuberin (EDFTEFV)**	Arg 211, Asn 216, Ser 219	Ala 89, Ile 221, ser 218, Arg 299,Glu 296, Glu 292, Gly 153	−1.883
**Control Peptide (KNNPNNAHQN)**		**Did not dock with HtrA2**	

The possible residues which are involved in hydrogen bonding and Vander Waal’s interactions along with Glide scores are mentioned.

**Figure 4:**
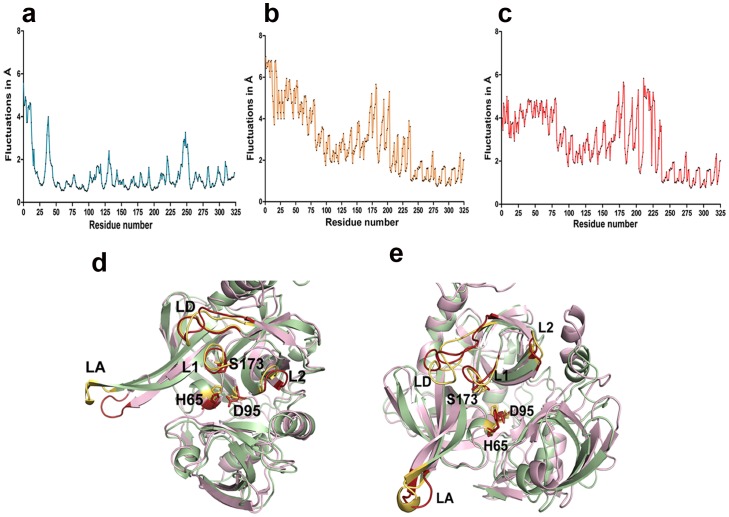
Graphical representation of root mean square fluctuation (RMSF) and loop movements upon peptide binding. a. MD simulation trajectory for unbound HtrA2. b. RMSF graph for GQYYFV bound HtrA2. c. RMSF graph for GSAWFSF bound HtrA2. d. Comparison of fluctuations in loops LA, L1, L2 and LD in the GQYYFV peptide bound (pink) and unbound structure (green). The loops in the bound and unbound forms are displayed in red and yellow respectively. e. Comparison of fluctuations in loops LA, L1, L2 and LD in the GSAWFSF peptide bound (pink) and unbound structure (green). The loops in the bound and unbound forms are displayed in red and yellow respectively. The catalytic triad residues are shown in both panels d. and e.

The RMSF of these trajectories were comparable with rmsd values showing higher relative fluctuations in and around the hinge region. Representative RMSF plots for GQYYFV and GSAWFSF bound HtrA2 complexes depict these large fluctuations for residues 190–225 as shown in Figures 4b and C respectively. All structural alignment comparisons and relative fluctuation analyses post MDS emphasize distinct significant conformational change in the hinge (211–226) region upon peptide binding. In addition to this, binding of peptides led to dynamic movements in many functionally important regions distal to SBP such as helices α5 and α7 in PDZ domain.

### Conformational Transitions in Flexible Regions and at the Active Site

Further detailed analyses of the effect that local subtle structural changes at SBP had on distal regions of the protease especially at the active site and its vicinity revealed the possibility of SBP being a putative allosteric site. Functional active site formation and its accessibility along with a well formed oxyanion hole are important prerequisites for the activity of an enzyme.

Structural comparison of the MD simulated peptide bound structure of HtrA2 with the unbound form show movements in different domains and linker regions. The PDZ-protease linker that covers the peptide binding groove in the PDZ domain moves away from it thus increasing it accessibility. The peptide bound HtrA2 complex show relative movements in the active site triad residues compared to the unbound form. Atomic distance analysis of both the forms revealed that distances between nitrogen (ε) atom of H65 and oxygen (γ) atom of S173 increased in peptide bound complexes while that between nitrogen (δ) atom of H65 and oxygen (δ) of D95 decreased when compared with the unbound HtrA2 structure (Table 3). This pattern being consistent with both the peptides suggests that interaction of peptide activator with SBP leads to opening up of the active site cleft.

**Table 3:** Comparison of distances between atoms of the catalytic triad in the peptide bound and unbound forms of HtrA2.

Protein Complex	NE2 (His) – OG (Ser)		ND1 (His) – OD1(Asp)	
	Bound	Unbound	Bound	Unbound
HtrA2 (GSAWFSF)	5.2	4.1	2.6	2.9
HtrA2 (GQYYFV)	5.5	4.1	2.7	2.9

Apart from active site triad, changes were also observed in the orientation of mechanistically important L1, LD and LA loops in the peptide bound complex (Figures 4d–e). Their orientations with respect to the active site determine proper oxyanion hole formation, accessibility of the active site, formation of catalytic triad and hence enzyme activity. MDS analyses for these regions showed significant deviations upon peptide binding. Structural alignment of GSAWFSF bound HtrA2 complex with the unbound form demonstrated breaking of Van der Waals contacts between loop LD and β2 strand of protease domain which facilitates LD movement towards α1 of protease domain and bringing P130 of the former in proximity to A25 of the latter. Similarly, S50 in β2 of protease domain establishes interactions with G171 of L1 (oxyanion hole residue) while breaking contacts with A132 of LD loop due to movement or tilt in the L1 loop. As a result of this reorganization, LD which was closer to L1 in the unbound HtrA2 moves sharply away from it upon peptide binding. These positional rearrangements also lead to disruption of interaction between D165 of L1 and G195 of L2 loops. All these movements coordinate to bring LD closer to the proximal region of protease domain thereby opening up the catalytic site. For GQYYFV peptide, movements of all these loops were subtle as compared to that for GSAWFSF except for the LA loop which exhibited larger deviation in the former. The other significant flexible region movement is in loop L3 which, in concert with linker region, assists in accommodating the peptide at SBP.

The relative reorientation of these loops along with catalytic triad residues seems to be assisting formation of a more open structure near the active site. However, loop L2 that harbors the specificity pocket remains mostly unchanged suggesting presence of a well formed binding pocket in the unbound form whose accessibility is limited compared to the substrate bound form. In context with trimeric HtrA2, more open conformation might be significant as it enhances the accessibility of the substrate and thereby might contribute positively toward the rate of enzyme catalysis.

### Influence of SBP on HtrA2 Activity and Role of PDZ Domain

To determine whether critical SBP residues (N216, S219, E292 and E296) are important for mediating allosteric propagation in HtrA2, site directed mutagenesis to alanine were done. Mutation of a conserved YIGV residue (G230A) was also done to understand the role of canonical YIGV groove in this complex signal propagation pathway. Moreover, since the protein is found to be active in its trimeric form [4] and also that SBP encompasses a major part of PDZ, we used trimeric and monomeric HtrA2 variants, N-SPD and F16D respectively to understand the role of PDZ in intra and inter-molecular cross-talk.

**Figure 5:**
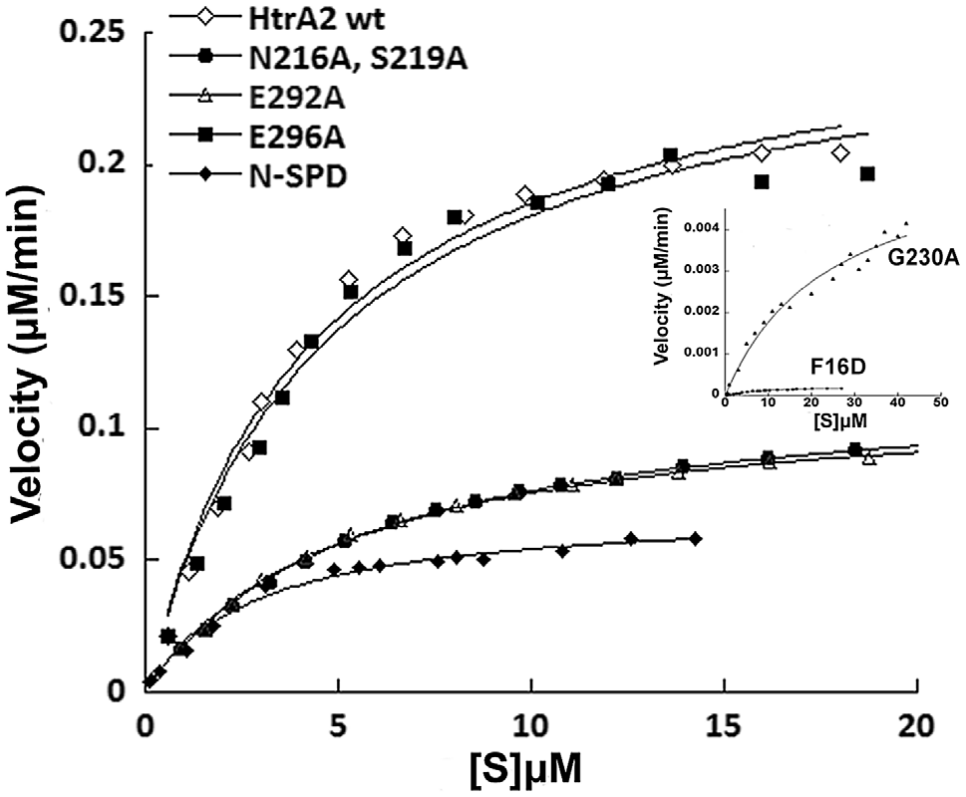
Steady state kinetic parameters of HtrA2. Graph representing relative activity of wild type HtrA2 and its mutants and variants with FITC labelled β-casein as the substrate. The graph for two mutants (F16D and G230A) is shown in inset.

To negate the role of overall conformational changes if any due to these mutations, MDS and secondary structural analyses were done on the mutant proteins. Similar active site conformations were observed in both the wildtype and mutants. Moreover, the overall secondary structure and thermal stability remained unperturbed due to the mutations (data not shown). Enzymology studies with different SBP mutants were done using β-casein, a well-established generic substrate of serine proteases [23]. β-casein has a putative SBP binding site (GPFPIIV) which has been found to interact with the similar residues at SBP by our docking studies (Table 2) and hence expected to mimic the allosteric modulation mediated by SBP binding if any**.** The kinetic parameters for wild type, N-SPD domain, F16D and other mutants were determined using fluorescent β-casein (Figure 5). The catalytic efficiency (k_cat/_K_m_) for the double mutant N216A/S219A and single mutant E292A showed ∼2.4 fold decrease in enzyme activity as compared to wild type whereas enzymatic parameters remained mostly unchanged for E296A. K_m_ values for the mutants were not significantly higher compared to the wild type, suggesting that the specificity pocket might be mostly intact with some subtle alterations. However, there was a marked decrease in V_max_ and in substrate turnover (k_cat_) rates for N216A/S219A and E292A suggesting presence of a malformed oxyanion hole in the SBP mutants. These results demonstrate that N216/S219 and E292 of SBP are important for mediating allosteric activation of HtrA2 upon activator binding. This is strengthened by the observation that SBP mutants did not interact with the activating peptides as seen by isothermal calorimetric studies and a representative figure is shown in the supplementary material (Figure S3). In addition, the ligplot of the peptide showing the detailed interaction with HtrA2 is also depicted in figure S1.

In our in silico studies, YIGV has been found to be a part of the greater SBP mesh (Table 1) and since docking with small molecular fragments (∼35–100 Da) showed direct binding with YIGV residues (Table S1), we wanted to understand the effect of YIGV mutation on HtrA2 activity as well. Enzymology studies with G230A demonstrated increase in K_m_ value compared to the wild type highlighting the involvement of YIGV in this intricate allosteric mechanism. Protein turnover rate was also much lower in G230A as compared to the wild type reiterating the importance of oxyanion hole formation upon activator binding at SBP. Thus, inaccessibility of the canonical PDZ binding pocket YIGV, in the trimeric protease structure might have adjured presence of exposed SBP which is dynamically coupled to YIGV groove for efficient allosteric signal propagation to the distal active site. Direct binding of small molecules at YIGV supports this hypothesis as they could be accommodated in the classical binding groove without requirement of any initial conformational change as it might be with the larger peptide activators.

Interestingly, although catalytic efficiency for N-SPD has been found to be 3.4 fold less as compared to the wildtype, its K_m_ value suggests slight increase in substrate affinity for the enzyme (Table 4). This increase in substrate affinity might be due to absence of PDZ surrounding the active site region resulting in greater substrate accessibility. However in N-SPD, k_cat_ was found to be 5 fold less than that of wild type highlighting the role of PDZ in initiating conformational changes near the active site pocket as well as in the oxyanion hole so as to increase overall enzyme stability. However, in the full length monomeric mutant of HtrA2 (F16D), there is a two fold increase in K_m_ with significant decrease in turnover rate and hence catalytic efficiency (Table 4) which emphasizes importance of intermolecular crosstalk between PDZ and protease domains in trimeric HtrA2 structure.

**Table 4:** Steady state kinetic parameters for HtrA2 wild type, variants and mutants with β-casein as the substrate.

*HtrA2* *Proteins*	*K_m_ (µM)*	*V_max_ (M/s)*	*k_cat_ (1/s)*	*k_cat_/K_m_* *(1/M.s)*
**Wild type**	4.59	4.083×10^−9^	0.02041	4.452×10^3^
**N216A, S219A**	5.43	1.937×10^−9^	0.00968	1.788×10^3^
**E292A**	5.15	1.903×10^−9^	0.00951	1.849×10^3^
**E296A**	4.68	3.734×10^−9^	0.01868	3.995×10^3^
**N-SPD**	3.02	0.7851×10^−9^	0.0039	1.29×10^3^
**F16D**	9.3	4.08×10^−12^	0.000025	0.0026×10^3^
**G230A**	9.32	1.03×10^−9^	0.0051	0.54×10^3^

**Figure 6:**
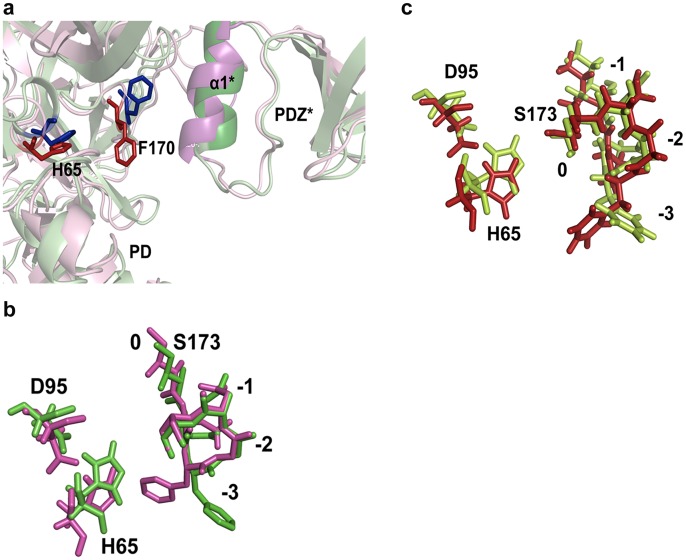
Structural changes at the oxyanion hole and YIGV groove upon peptide binding. a. Overlay of the oxyanion hole and catalytic triad residues represented as sticks for peptide GQYYFV bound (magenta) and unbound (green) structures. PD denotes serine protease domain of HtrA2. b. Overlay of the oxyanion hole and catalytic triad residues represented as sticks for peptide GSAWFSF bound (red) and unbound (limon green) structures. c. Role of PDZ in the formation of proper active site formation. The structural superposition of GQYYFV bound (pink) and unbound (green) structures shows α5 helix of PDZ of one subunit moves towards the LD loop and oxyanion hole of the adjacent subunit. The positions of the residues in the oxyanion hole are denoted as 0, −1, −2 and −3.

The importance of intermolecular interaction between PDZ* and SPD has also been manifested in our MD studies where structural analyses show binding of peptide activator (GQYYFV) at the SBP alters PDZ orientation and brings α5 helix of PDZ from one subunit in close proximity to the protease domain of the adjacent subunit. The helix moves towards LD loop of the protease domain, thereby shifting the orientation of the phenyl ring of F170 which is a part of oxyanion hole towards H65 of the catalytic triad (Figure 6a) so as to accommodate the loop. These rearrangements result in a more stable and catalytically competent HtrA2 formation with a proper oxyanion hole. Thus the full length trimeric HtrA2 is more active than trimeric N-SPD, where the activation pocket is not stable in absence of PDZ.

## Discussion

Our aim was to understand the structural dynamics that regulates activation and specificity of HtrA2. This multidomain trimeric protease has unique proapoptotic properties as it is associated with both caspase-dependent and independent cell death pathways through its serine protease activity [5], [12]. Association of HtrA2 with cancer and neurodegenerative disorders makes it a promising therapeutic target. For example, overexpression of HtrA2 substrates such as IAPs and the Wilms’s tumor suppressor protein WT1 in several cancers suggests modulation of HtrA2 protease activity can effectively regulate their relative levels in the cells [24], [25], [26], [27]. Out of several approaches that can be used to regulate HtrA2 activity, allosteric modulation is one of the simplest and most efficient ways. However, modulating HtrA2 functions with desired characteristics for disease intervention will require a detailed understanding of its mode of activation and the underlying conformational plasticity that controls it.

Peptide design using site complementarity followed by MDS of the docked peptide-macromolecular complex is an extremely useful tool to study subtle conformational changes and protein dynamics. HtrA2 has a complex network of flexible loops surrounding the active site pocket and a linker at the PDZ-protease interface whose relative orientations and crosstalk with different domains might be critical in defining HtrA2 functions. With partially missing loops and the flexible linker region, the solved structure of HtrA2 [4] could not fully explain the dynamics and allostery that regulate its activity and specificity. Here, with an *in silico* and biochemical approach, we have shown that like few other HtrA family proteins, allosteric propagation does regulate HtrA2 activity.

In this study, peptide binding to SBP showed conformational changes in the distal flexible regions of HtrA2 such as the PDZ-protease interface, loops L1, LD and LA that rearrange to form a more catalytically efficient active site thus establishing the role of SBP as an allosteric site in HtrA2. A close look at and around the active site pocket shows that in the bound form, the N atom of Gly (−2 position) faces the oxyanion hole to form an H-bond whereas in the unbound form it flips in the opposite direction to form a malformed oxyanion hole [12], [28]. Moreover, keeping in trend with other HtrA proteases, the phenylalanine ring of −3 position moves closer to the imidazole ring of His65 while in the unbound form, it moves outward as observed from Figures 6b–c and Movie S1. All these subtle structural rearrangements along with making and breaking of bonds at sites away from the active site might stabilize the peptide bound form such that it shifts the equilibrium toward catalysis.

Enzymology studies with β-casein that has a putative SBP binding sequence (GPFPIIV) as shown in Table 4 show significant decrease in catalytic efficiency in SBP mutants. This observation suggests interaction of substrate protein with SBP brings about rearrangement around the active site of the enzyme by positively influencing its activity thus behaving as an allosteric regulator. The SBP mutants (N216A/S219A and E292A) show apparent decrease in V_max_ without significantly altering the apparent K_m_ (with L2 specificity pocket mostly unaltered) and hence follow the ‘V system’ of allosteric modulation [29]. In this system, both the relaxed (R) and the tensed (T) states bind the substrate at the active site with similar affinity while the peptide (activator) at SBP binds the R and T states with different affinity. This differential affinity of the peptide towards SBP along with R state stabilization shifts the equilibrium towards R state thus positively influencing its turnover rate and hence catalytic efficiency which has been observed in case of HtrA2.

In N-SPD, where the PDZ domain is absent, apparent decrease in K_m_ can be attributed to greater accessibility of the substrate to the active site. However, since the change in binding affinity is not large, the specificity pocket might be mostly unaltered compared to the wild type which is confirmed through our MD studies where the loop L2 remains mostly unaltered. Interestingly, k_cat_ value in N-SPD has been found to decrease significantly which is suggestive of either a malformed oxyanion hole and/or decrease in overall protein stability which might be due to absence of supporting PDZ domain. However, similar studies with F16D (monomeric full length HtrA2 mutant) also show significant decrease in turnover rate and catalytic efficiency which accentuates the importance of intermolecular and not intramolecular PDZ-protease crosstalk in trimeric HtrA2. Our MDS supports this observation by demonstrating that in the peptide bound form of HtrA2, α5* of PDZ* moves towards LD loop of protease domain of adjacent subunit thus pushing phenyl ring of F170 of the oxyanion hole towards H65 of the catalytic triad (Figure 6a). This reorientation in the oxyanion hole makes the protease poised for catalysis as seen in other HtrA family members as well [12] thus significantly enhancing the turnover rate. Therefore, intermolecular crosstalk stabilizes the active site and makes it catalytically competent establishing the requirement of complex trimeric architecture of the protease.

**Figure 7:**
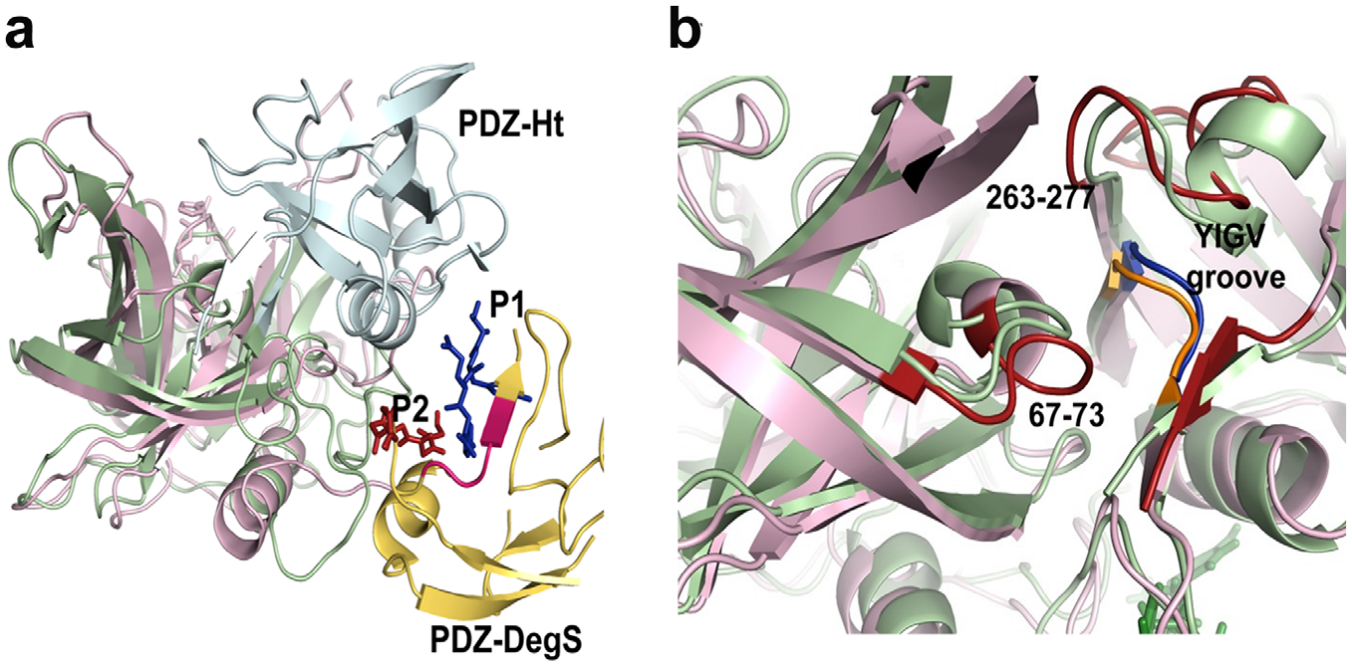
Structural comparison of PDZ domain orientation. a. Structural alignment of *E.coli* DegS (PDB ID: 1SOZ) and the peptide bound HtrA2 showing PDZ domains for both the proteins (represented in blue and yellow respectively) are oriented differently but the peptides, P1 (blue) and P2 (pink) represented as sticks for the respective proteins seem to bind to a structurally similar region. The GLGF substrate binding motif is exposed for DegS while buried for HtrA2 as shown in pink and blue respectively. b. Alignment of the peptide bound (pink) and unbound (green) structures at the region around the YIGV groove shows outward movement of the loops spanning residues 67–73 and 263–277 shown in red for the bound structures which leads to opening up of the YIGV groove.

The GLGF motif (YIGV in HtrA2) is the canonical peptide binding site [2], [4] in PDZ domains. However, in HtrA2, it is deeply embedded within a hydrophobic groove where the residues are intertwined with each other through several intramolecular interactions making the site highly inaccessible to the binding of peptide [4]. Thus, peptide binding to YIGV is only possible upon certain structural rearrangements at that site. Given the property of PDZ domains of having multiple docking sites and the fact that HtrA2 requires huge conformational changes for proper active site formation, we hypothesized presence of a relatively exposed pocket where peptide binding occurs prior to interaction with the buried YIGV groove. In our studies, we have found a novel surface exposed region (SBP) around PDZ domain which is easily accessible to the peptide. With an aim at understanding the allosteric mechanism in HtrA2 and whether the binding site is structurally conserved, we did a side-by-side comparison with the peptide-bound PDZ structure of its bacterial counterpart DegS that is known to exhibit allostery [30]. The structural overlay of peptide bound forms of these two proteins show striking structural similarity in the regions of binding (Figure 7a) with the GLGF groove (YIGV in HtrA2 and YIGI in DegS) oriented differently. Since the YIGV motif is buried in HtrA2 structure, its inaccessibility might be the reason for the peptide to initially bind to another relatively accessible region with similar hydrophobic milieu. However, in DegS, the YIGI groove is already exposed to accommodate the peptide easily and hence this kind of initial interaction is not required.

**Figure 8:**
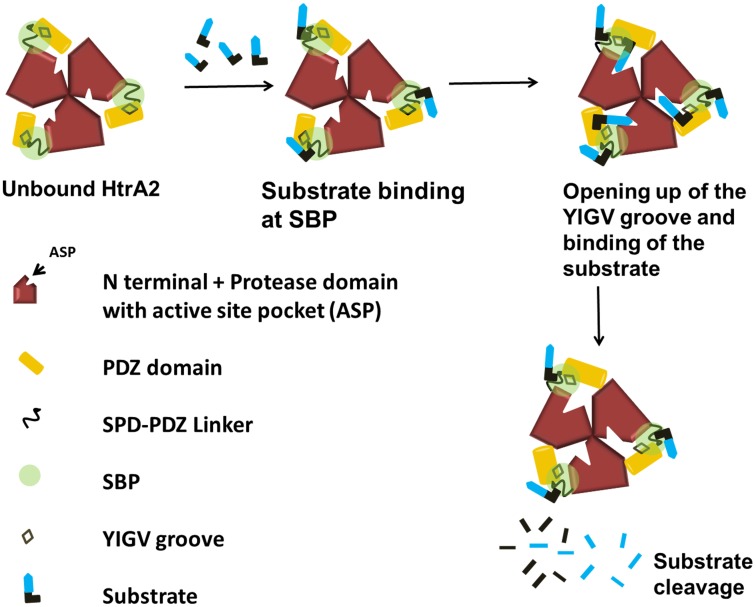
Allosteric model for HtrA2 protease activity. The substrate protein binds to relatively exposed part of SBP due to inaccessibility of the YIGV groove which triggers opening up of the PDZ domain. This reorientation makes the YIGV groove accessible for substrate interaction and the PDZ of a subunit moves closer to the protease domain of the adjacent subunit leading to formation of a proper active site and oxyanion hole. This complex allosteric signal propagation leads to subsequent substrate binding and catalysis at the active site pocket. Thus structural perturbations at these two distant sites (SBP and catalytic pocket) might be dynamically coupled to the canonical peptide binding groove through a complex allosteric mechanism.

Our MDS studies show that peptide binding at SBP leads to subtle structural changes in the region adjoining YIGV leading to opening up of the pocket. The last β strand of PDZ domain which lies on one side of YIGV groove moves away from it. The YIGV and the loop spanning residues 67–73 move away from each other while the loop comprising residues 263–277 of the β-α-β motif also drifts at an angle away from the YIGV making it more solvent exposed (Figure 7b). Therefore, upon SBP binding, the relative movements of the loops in vicinity of the hydrophobic YIGV pocket might confer it with the kind of exposure that is required for interaction with peptides. These observations along with our enzymology studies with SBP and YIGV mutants, led to defining a model (Figure 8) for allosteric propagation in HtrA2. The model suggests that initial binding of the peptide activator at SBP leads to structural fluctuations which result in subtle rearrangement at and around the YIGV groove (a part of greater SBP mesh as identified by Sitemap) thus exposing it. Opening up of the deeply embedded YIGV pocket makes it accessible to the substrate molecule which consequently leads to allosteric signal propagation at the active site in the serine protease domain.

This alternative non-canonical PDZ binding site though novel in HtrA family of proteins, is not unprecedented in literature. It has been observed that PDZ7 of the scaffold protein Glutamate receptor interacting protein 1 (GRIP1) has an alternative exposed hydrophobic pocket that binds its substrate GRASP-1 since the canonical binding site is deeply embedded within the protein [31]. Overlay of the PDZ from HtrA2 and PDZ7 of GRIP1 show striking structural similarity including the classical peptide binding groove and the novel non-canonical pocket (Figure S2). Thus, in these two proteins, perturbations at the alternative distal binding sites might be coupled dynamically to the classical binding groove by a complex mechanism that includes fast (ps–ns) timescale dynamics which consequently leads to allosteric signal propagation to the active site.

In the recent past, allosteric modulators have evolved into important drug targets due to several advantages they have over orthosteric ligands that include more diversity, less toxicity and absolute subtype selectivity [32], [33]. Therefore, designing suitable SBP binding peptides or peptidomimetics of HtrA2 might be an excellent approach to modulate HtrA2 functions for devising therapeutic strategies against various diseases it is associated with.

## Materials and Methods

### Loop Modeling and Site Prediction

Crystal structure of HtrA2 (PDB ID: 1LCY) [4] obtained from Protein Data Bank [34] has missing N-terminal residues (AVPSP) and two flexible regions (^211^RGEKKNSSSGISGSQ ^225^ and ^149^ARDLGLPQT ^157^). These missing structures were modelled and loops were refined using Prime 3.0 (Schrödinger, LLC, New York, 2011). which was later subjected to molecular dynamics simulation for 5 ns with GROMACS, version 4.5.1 [17] to obtain the lowest energy structure of HtrA2. The binding sites were then predicted using SiteMap 2.5 (Schrödinger, LLC, New York, 2011). Out of 5 pockets predicted, the site that scored the best based on its size, hydrophobic and hydrophilic characters, degree to which ligand might donate or accept hydrogen bonds and exposure to solvent was selected for further analysis. This site selective binding pocket (SBP) encompasses PDZ-protease interface with the involvement of hinge region and a part of PDZ domain (Table 1).

### Peptide Designing and Molecular Docking

Based on properties of amino acids lining the binding site, fragment docking (Glide XP, Schrödinger, LLC, New York, 2011) [35] approach was used to dock 20 amino acids and 8 functional group replicas (N-methylacetamide, methanol, phenol, benzene, propane, acetate ion, methylammonium, methylguanidinium) at SBP [36]. Based on properties of the amino acids that form SBP, replicas were chosen and were used for generating fragments in combinations of four as shown in Table S1. Combine Fragment tool (Schrödinger, LLC, New York, 2011) was used to join the fragments which were docked at SBP with three major filtering options (bond angle deviation 5 degrees, atom-atom distance 1 Å and fragment centroid distance 2.0). The set of replica functional groups that displayed the best docking scores were used to build the peptide. The amino acids Arg, Ser, Gln, Glu, Asp, Asn, Thr, Lys and their positions in tetrapeptide combination were chosen based on the functional groups they resembled. Subsequently all possible peptide combination of these amino acids with respect to their relative positions were generated. The predicted tetrameric peptides (Table S2) were selected and docked again with SBP.

In parallel, another mode of designing was used by identifying signature peptides from literature which bind HtrA2 [19]. Initially two peptides were chosen (GQYYFV and GGIRRV) and based on the sequence similarity and hydrophobicity, stretches of putative binding residues from two known binding partners of HtrA2 were identified (GPFPIIV from C-terminal region of β-casein and GSAWFSF, an internal motif of antiapoptotic Pea-15) [37]. A putative HtrA2 binding pattern was designed based on phage display library [21] which along with the earlier four sequences was used to generate all possible peptide combinations**.** Considering structural complementarity and three dimensional arrangements of amino acids at SBP, Gly and Val residues were added at N- and C-termini of some peptides to increase the stability of the docked complex. A 10 mer peptide having the sequence KNNPNNAHQN that does not match the consensus SBP binding peptide pattern was used as a negative control. These combinations were used for searching all possible sequences of known and potential HtrA2 binding partners [38].

All designed peptides were built *in silico* using BREED (Schrödinger, LLC, New York, 2011) and Combine Fragments tools which were then prepared for docking using LigPrep 2.5 (Schrödinger, LLC, New York, 2011). After ligand preparation, Confgen 2.3 (Schrödinger, LLC, New York, 2011) was used to generate all possible energetically minimum conformers of the designed peptides which were then docked using Glide [39], [40].

In the modeled HtrA2 structure, energy minimization was done using Protein Preparation Wizard 2.2 (Epik Version 2.2, Schrödinger, LLC, New York, 2011) after addition of H-atoms. Molecular Docking was initiated by preparing Grid file (input file) which contains receptor (protein structure) and binding site information (Prime output). All three precision methods which include high throughput virtual screening (HTVS), standard precision (SP) and extra precision (XP) [35] of Glide [39], [40] were used for docking these peptides on SBP. This series of docking methods were used to filter out energetically less favorable peptide conformers and get a subset of best possible peptides for further studies.

### MD Simulation (MDS) and Analysis

After analyzing the docking results, best HtrA2-peptide complexes based on Glide XP score and E-model value were used for Molecular Dynamic Simulation which was performed using Desmond 2010 [22] software package. Optimized Potentials for Liquid Simulations (OPLS) [41] all-atom force field was used to analyze model stability. The protein structures were solvated with Monte Carlo simulated TIP3P [42] water model with a 10 Å buffer space from the protein edges in an orthorhombic box and the system was then neutralized by replacing water molecules with sodium and chloride counter ions. Similarly, unbound HtrA2 system was also developed as a control. Neutralization of systems was done by adding 2 Na^+^ ions in unbound HtrA2 and 4 Na^+^ ions for peptide bound complexes. The particle-mesh Ewald method (PME) [43] was used to calculate long-range electrostatic interactions with a grid spacing of 0.8 Å. Van der Waals and short range electrostatic interactions were smoothly truncated at 9.0 Å. Nose–Hoover thermostats were utilized to maintain the constant simulation temperature and the Martina–Tobias–Klein method was used to control the pressure [44]. The equations of motion were integrated using the multistep RESPA integrator [45] with an inner time step of 2.0 fs for bonded interactions and non-bonded interactions within the short range cut-off. An outer time step of 6.0 fs was used for non-bonded interactions beyond the cut-off. These periodic boundary conditions were applied throughout the system.

These prepared systems were equilibrated with the default Desmond protocol that comprises a series of restrained minimizations and MDS. Two rounds of steepest descent minimization were performed with a maximum of 2000 steps and a harmonic restraint of 50 kcal/mol/per Å^2^ on all solute atoms followed by a series of four MDS. The first simulation was run for 12 ps at a temperature of 10 K in the NVT (constant number of particles, volume, and temperature) ensemble with solute heavy atoms restrained with force constant of 50 kcal/mol/Å ^2^. The second simulation was similar to the first except it was run in the NPT (constant number of particles, pressure, and temperature) ensemble. A 24 ps simulation followed with the temperature raised to 300 K in the NPT ensemble and with the force constant retained. The last one was a 24 ps simulation at 300 K in the NPT ensemble with all restraints removed. This default equilibration was followed by a 5000 ps NPT simulation to equilibrate the system. A 30 ns NPT production simulation was then run and coordinates were saved in every 2 ps of time intervals.

The total trajectory of MD simulation was 30 ns. MD Simulation was analyzed using the analytical tools in the Desmond package. In MD quality analysis, potential energy of the protein as well as total energy of the entire system was calculated. The lowest potential energy conformations were then used for comparative analysis of peptide bound and unbound structures. Trajectories of peptide bound complexes and unbound HtrA2 were then compared based on their overall calculated RMSD (root mean square deviation), domain wise RMSD and RMSF (root mean square fluctuation) values and were plotted using GraphPad Prism 5.0 (GraphPad Software, San Diego, CA, USA).

### Production of Recombinant HtrA2 Wild Type, its Mutants and Domains

Mature (Δ133 HtrA2) with C-terminal his_6_-tag in pET-20b (Addgene, Cambridge, MA) was expressed in *E. coli* strain BL21 (DE3) pLysS. N-SPD, comprising N-terminal and serine protease domains (residues 1–210) of HtrA2 was sub cloned into pMALc5E-TEV using appropriate primers. Point mutations were introduced into pET-20b Δ133 HtrA2 by PCR using primer sets that included mutations for residues N216A, S219A, E292A, E296A and F16D. N-SPD clone and these mutants were confirmed by DNA sequencing. Protein expression was induced by culturing cells at 18°C for 20 h in presence of 0.2 mM isopropyl-1-thio-D-galactopyranoside. Cells were lysed by sonication and the centrifuged supernatants for HtrA2 and its mutants were incubated with pre-equilibrated nickel-IDA beads for 1 h at room temperature. Protein purification was done using Ni-affinity chromatography as described earlier [19]. Eluted protein was further purified using gel permeation chromatography. N-SPD was purified using amylose resin where the bound protein was eluted using 10 mM maltose and was subjected to TEV protease cleavage [46] to remove maltose binding protein (MBP). N-SPD was further separated from MBP by gel filtration using Superdex 75 column. All purified proteins were analyzed by SDS-PAGE for purity. The fractions with >95% purity were stored in aliquots at −80°C until use.

### FITC-β-Casein Cleavage Assay

The proteolytic activity of wild type and the mutants were determined using FITC-labelled β-casein cleavage assay [47]. Fluorescent substrate cleavage was determined by incubating 200 nM of enzymes with increasing concentration (0–25 µM) of β-casein at 37°C in cleavage buffer (20 mM Na_2_HPO_4_/NaH_2_PO_4_, pH 8.0, 100 mM NaCl, 0.1 mM DTT). Fluorescence was monitored in a multi-well plate reader (Berthold Technologies) using excitation wavelength of 485 nm and emission at 545 nm. Reaction rates v_0_ (µM/min) were determined by linear regression analysis corresponding to the maximum reaction rates for individual assay condition. Assays are representative of at least three independent experiments done in triplicate. The steady-state kinetic parameters were obtained from the reaction rates by fitting data to Michaelis-Menten equation using nonlinear least squares subroutine in KaleidaGraph program (Synergy software).

## Supporting Information

Figure S1 **Interaction of peptides with HtrA2.** a. Ligplot for GSAWFSF with HtrA2 which represents residues involved and the nature of interactions. b. Ligplot for GQYYFV interaction pattern with HtrA2. c. Ligplot for GPFPIIV with HtrA2 which represents residues involved and the nature of interactions. d. Ligplot for SEHRRHFPNCFFV peptide with HtrA2 which represents residues involved and the nature of interactions. The residues of peptides and HtrA2 involved in interaction are shown in blue and red respectively.
(TIF)

Figure S2 **Comparison of SBP and allosteric pocket of GRIP-1 protein.** Structural overlay of the protein GRIP-1(green) bearing PDB ID 1M5Z and GQYYFV bound HtrA2 (pink) shows striking resemblance of the orientation of buried GLGF motif shown in yellow and blue respectively. The α helix denoted as αB (green) for GRIP-1, known to be involved in formation of allosteric pocket overlays very well with the one involved in SBP formation (orange) in GQYYFV (red sticks) -HtrA2 complex.
(TIF)

Figure S3 **ITC studies for activating peptide with HtrA2 and the SBP double mutant.** The peptide used was 13mer SEHRRHFPNCFFV, which has similar consensus sequence as defined for PDZ peptide groove binding substrate. The peptide was better in terms of solubility as compared to other activating peptides and binding studies were done using Isothermal titration calorimetry. The titrations were carried out using Micro Cal ITC200 (GE Healthcare) with the calorimetry cell containing 200 *µ*l of wild type or N216A/S219A mutant HtrA2 in 20 mM Na_2_HPO_4_/NaH_2_PO_4_ buffer, 100 mM NaCl, pH 7.8. The concentration of protein was in range from 20 to 50 *µ*M and was titrated with 1.5 *µ*l injections of a solution containing 0.4 mM activator peptide reconstituted in the same buffer. To correct the effect of heat of dilution, a blank injection was made under identical conditions. All experiments were performed at 25°C and the data was analyzed using the manufacture provided MicroCal software with the integrated heat peaks fitted to a one site-binding model. Simulated ITC raw data for the protein with the activating peptide is represented in the upper panel and the integrated data in the lower panel. The dissociation constant was calculated to be 7.5* µ*M for wild type (left panel) and no significant heat change was observed for the SBP double mutant (right panel).
(TIF)

Table S1 **Docking analysis of replica fragments with HtrA2.** The fragments have been arranged according to their docking scores.
(DOC)

Table S2 **Designed peptide fragments.** Fragments of peptide combinations generated based on functional group studies have been enlisted.
(DOC)

Movie S1 **Orientation of active site triad and oxyanion hole formation during MD simulation of HtrA2-peptide complex.** From this visual representation of HtrA2 peptide (GSAWFSF) complex during MD simulation it can be seen that the catalytic triad residues H65, D95, S173 reorient to form an active conformation along with oxyanion hole residues (N172, G171 and F170). All the residues involved are represented as sticks. This movie shows proper active site and oxyanion hole formation.
(AVI)
