# Supplementary figures and images for: Allosteric Regulation of Serine Protease HtrA2 through Novel Non-Canonical Substrate Binding Pocket
Source: PLoS One. 2013 Feb 14;8(2):e55416. doi: 10.1371/journal.pone.0055416 (PMC3573033; doi:10.1371/journal.pone.0055416)

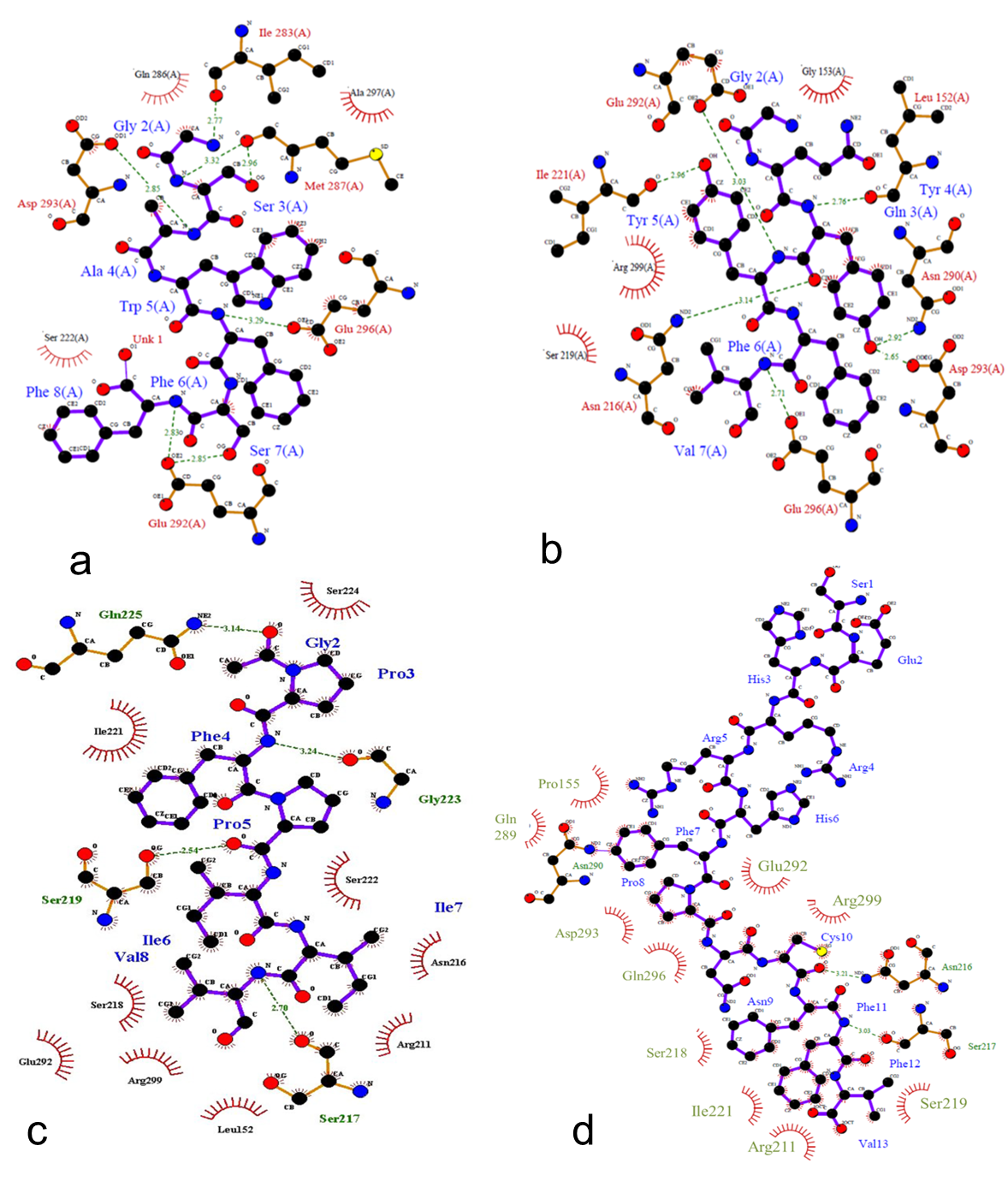

Supplement: Figure S1 — Interaction of peptides with HtrA2. a. Ligplot for GSAWFSF with HtrA2 which represents residues involved and the nature of interactions. b. Ligplot for GQYYFV interaction pattern with HtrA2. c. Ligplot for GPFPIIV with HtrA2 which represents residues involved and the nature of interactions. d. Ligplot for SEHRRHFPNCFFV peptide with HtrA2 which represents residues involved and the nature of interactions. The residues of peptides and HtrA2 involved in interaction are shown in blue and red respectively. (TIF) [file pone.0055416.s001.tif]

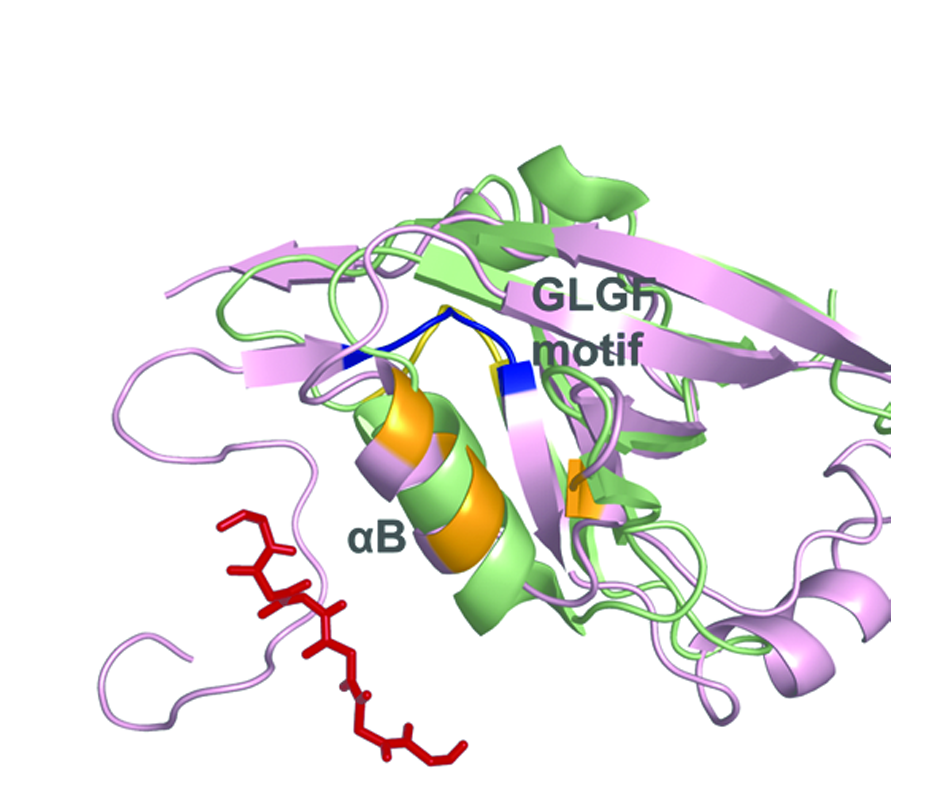

Supplement: Figure S2 — Comparison of SBP and allosteric pocket of GRIP-1 protein. Structural overlay of the protein GRIP-1(green) bearing PDB ID 1M5Z and GQYYFV bound HtrA2 (pink) shows striking resemblance of the orientation of buried GLGF motif shown in yellow and blue respectively. The α helix denoted as αB (green) for GRIP-1, known to be involved in formation of allosteric pocket overlays very well with the one involved in SBP formation (orange) in GQYYFV (red sticks) -HtrA2 complex. (TIF) [file pone.0055416.s002.tif]

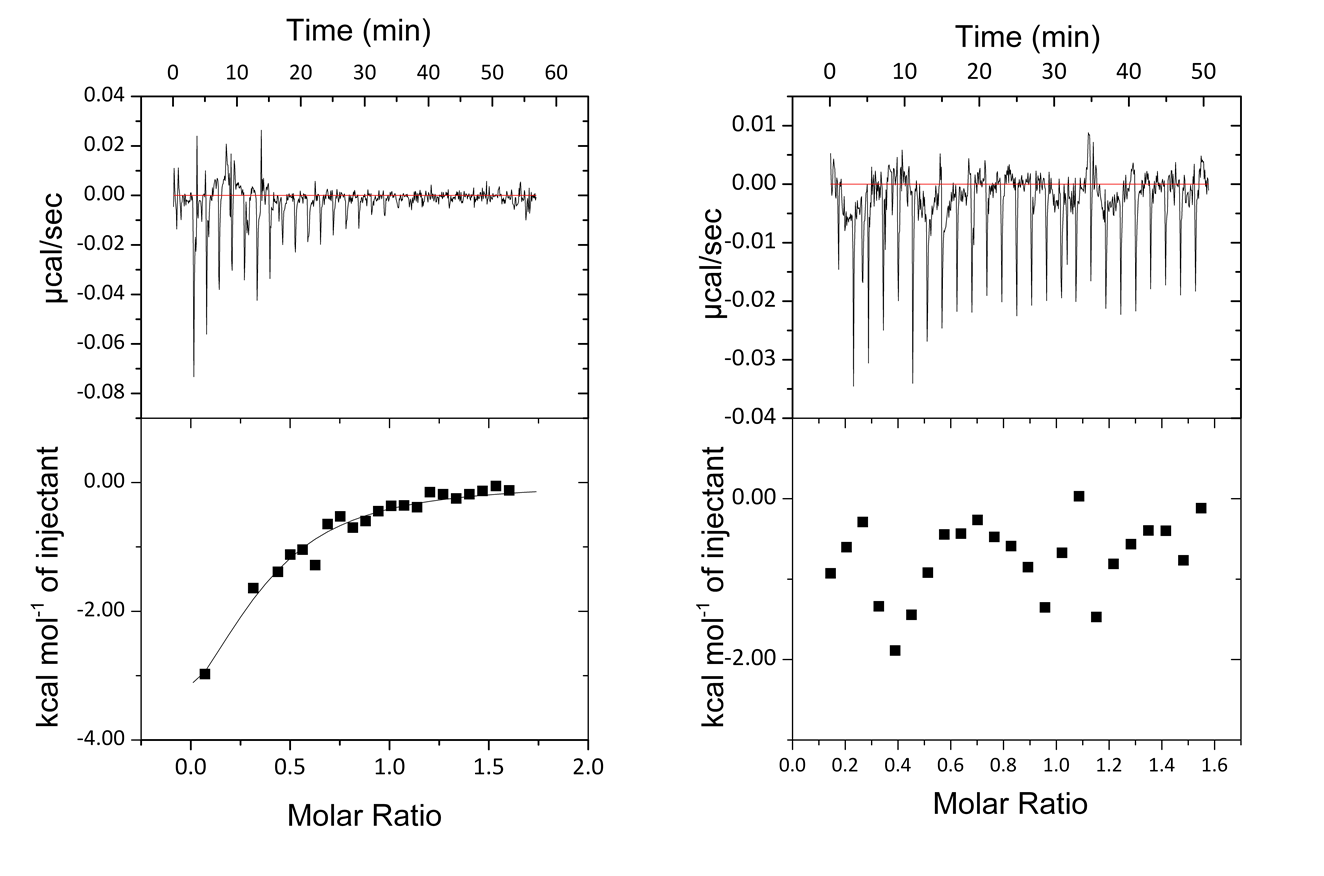

Supplement: Figure S3 — ITC studies for activating peptide with HtrA2 and the SBP double mutant. The peptide used was 13mer SEHRRHFPNCFFV, which has similar consensus sequence as defined for PDZ peptide groove binding substrate. The peptide was better in terms of solubility as compared to other activating peptides and binding studies were done using Isothermal titration calorimetry. The titrations were carried out using Micro Cal ITC200 (GE Healthcare) with the calorimetry cell containing 200 µl of wild type or N216A/S219A mutant HtrA2 in 20 mM Na2HPO4/NaH2PO4 buffer, 100 mM NaCl, pH 7.8. The concentration of protein was in range from 20 to 50 µM and was titrated with 1.5 µl injections of a solution containing 0.4 mM activator peptide reconstituted in the same buffer. To correct the effect of heat of dilution, a blank injection was made under identical conditions. All experiments were performed at 25°C and the data was analyzed using the manufacture provided MicroCal software with the integrated heat peaks fitted to a one site-binding model. Simulated ITC raw data for the protein with the activating peptide is represented in the upper panel and the integrated data in the lower panel. The dissociation constant was calculated to be 7.5 µM for wild type (left panel) and no significant heat change was observed for the SBP double mutant (right panel). (TIF) [file pone.0055416.s003.tif]
