# Supplementary material for: Allosteric Regulation of Serine Protease HtrA2 through Novel Non-Canonical Substrate Binding Pocket
Source: PLoS One. 2013 Feb 14;8(2):e55416. doi: 10.1371/journal.pone.0055416 (PMC3573033; doi:10.1371/journal.pone.0055416)
Supplement: Table S1 — Docking analysis of replica fragments with HtrA2. The fragments have been arranged according to their docking scores. (DOC) [file pone.0055416.s004.doc]

| **Fragment Designed** | **Residue interactions** | **Glide score** |
| --- | --- | --- |
| methylammonium - methanol :: acetate_ion – methanol | G230, V231, I229, D95, P92, Y295 | -7.092 |
| methylammonium - methanol :: acetate_ion – methanol | D95, V231 | -6.427 |
| methylammonium - acetate ion :: methanol – methanol | P92, A64, G230, V231 | -6.329 |
| N-methylacetamide - methanol :: methanol – methanol | D95, R226, V231 | -6.108 |
| methylammonium - methylammonium :: methanol – methanol | D95, R226, V231 | -5.870 |
| methylguanidinium - methanol :: methanol – methanol | D95, Y295, V231 | -5.850 |
| N-methylacetamide - methanol :: methanol – methanol | Y295, V231, R227, R226, D95 | -5.627 |
| methylguanidinium - methanol :: methanol – methanol | D95, Y295, V231 | -4.629 |
| methylammonium :: N-methylacetamide :: methanol – methanol | D95, R226, G230, V231 | -2.581 |
| methylammonium :: methanol :: methylguanidinium – methanol | D95, R226, G230, V231 | -1.804 |
| N-methylacetamide – methylammonium :: methanol – methanol | D95, V231 | -1.071 |

**Table S1.** **Docking analysis of replica fragments with HtrA2**. The fragments have been arranged according to their docking scores
