# Supplementary material for: Allosteric Regulation of Serine Protease HtrA2 through Novel Non-Canonical Substrate Binding Pocket
Source: PLoS One. 2013 Feb 14;8(2):e55416. doi: 10.1371/journal.pone.0055416 (PMC3573033; doi:10.1371/journal.pone.0055416)
Supplement: Table S2 — Designed peptide fragments. Fragments of peptide combinations generated based on functional group studies have been enlisted. (DOC) [file pone.0055416.s005.doc]

**Table S2.** **Designed peptide fragments.** Fragments of peptide combinations generated based on functional group studies have been enlisted

**Fragment1 combinations:**

Lys-Ser-Glu-Ser

Lys-Ser-Glu-Thr

Lys-Ser-Asp-Ser

Lys-Ser-Asp-Thr

Lys-Thr-Glu-Ser

Lys-Thr-Asp-Ser

Lys-Thr-Glu-Thr

Lys-Thr-Asp-Thr

**Fragment2 combinations :**

Arg-Ser-Glu-Ser

Arg -Ser-Glu-Thr

Arg -Ser-Asp-Ser

Arg -Ser-Asp-Thr

Arg -Thr-Glu-Ser

Arg -Thr-Asp-Ser

Arg -Thr-Glu-Thr

Arg -Thr-Asp-Thr

**Fragment3 combinations:**

Lys-Glu-Ser-Ser

Lys-Glu-Ser-Thr

Lys-Glu-Thr-Ser

Lys-Glu-Thr-Thr

Lys-Asp-Ser-Ser

Lys-Asp-Ser-Thr

Lys-Asp-Thr-Ser

Lys-Asp-Thr-Thr

Arg-Glu-Ser-Ser

Arg -Glu-Ser-Thr

Arg -Glu-Thr-Ser

Arg -Glu-Thr-Thr

Arg -Asp-Ser-Ser

Arg -Asp-Ser-Thr

Arg -Asp-Thr-Ser

Arg -Asp-Thr-Thr

**Fragment4 combinations:**

Gln-Ser-Ser-Ser

Gln-Thr-Ser-Ser

Gln-Ser-Thr-Ser

Gln-Ser-Ser-Thr

Gln-Thr-Thr-Thr

Asn-Ser-Ser-Ser

Asn-Thr-Ser-Ser

Asn-Ser-Thr-Ser

Asn-Ser-Ser-Thr

Asn-Thr-Thr-Thr

**Fragment 5 combinations:**

Lys-Lys-Ser-Ser

Lys-Lys-Thr-Ser

Lys-Lys-Ser-Thr

Lys-Lys-Thr-Thr

**Fragment 6 combinations:**

Arg-Ser-Ser-Ser

Arg -Thr-Ser-Ser

Arg -Ser-Thr-Ser

Arg -Ser-Ser-Thr

Arg -Thr-Thr-Thr

**Fragment 7 combinations:**

Gln-Ser-Ser-Ser

Gln-Thr-Ser-Ser

Gln-Ser-Thr-Ser

Gln-Ser-Ser-Thr

Gln-Thr-Thr-Thr

Asn-Ser-Ser-Ser

Asn-Thr-Ser-Ser

Asn-Ser-Thr-Ser

Asn-Ser-Ser-Thr

Asn-Thr-Thr-Thr

**Fragment 9 combinations:**

Lys-Asn-Ser-Ser

Lys-Asn-Thr-Ser

Lys-Asn-Ser-Thr

Lys-Gln-Thr-Ser

Lys-Gln-Ser-Thr

Lys-Gln-Thr -Thr

**Fragment 10 combinations:**

Lys -Ser- Arg-Ser

Lys -Thr- Arg-Ser

Lys -Ser- Arg-Thr

Lys -Thr- Arg-Thr

Arg -Ser- Arg-Ser

Arg -Thr- Arg-Ser

Arg -Ser- Arg-Thr

Arg -Thr- Arg-Thr
